# An on-site adaptable test for rapid and sensitive detection of *Potato mop-top virus*, a soil-borne virus of potato (*Solanum tuberosum*)

**DOI:** 10.1371/journal.pone.0270918

**Published:** 2022-08-01

**Authors:** Ying Zhai, Bryant Davenport, Keith Schuetz, Hanu R. Pappu

**Affiliations:** 1 Department of Plant Pathology, Washington State University, Pullman, WA, United States of America; 2 Agdia, Inc., Elkhart, IN, United States of America; University of Georgia, UNITED STATES

## Abstract

*Potato mop-top virus* (PMTV) is considered an emerging threat to potato production in the United States. PMTV is transmitted by a soil-borne protist, *Spongospora subterranean*. Rapid, accurate, and sensitive detection of PMTV in leaves and tubers is an essential component in PMTV management program. A rapid test that can be adapted to in-field, on-site testing with minimal sample manipulation could help in ensuring the sanitary status of the produce in situations such as certification programs and shipping point inspections. Toward that goal, a rapid and highly sensitive recombinase polymerase amplification (RPA)-based test was developed for PMTV detection in potato tubers. The test combines the convenience of RPA assay with a simple sample extraction procedure, making it amenable to rapid on-site diagnosis of PMTV. Furthermore, the assay was duplexed with a plant internal control to monitor sample extraction and RPA reaction performance. The method described could detect as little as 10 fg of PMTV RNA transcript in various potato tissues, the diagnostic limit of detection (LOQ) similar to that of traditional molecular methods.

## Introduction

Potato mop-top virus (PMTV), a member of the genus *Pomovirus* and family *Virgaviridae*, is an economically important virus of potato in many potato-producing regions in the world. The virus primarily infects plants in *Solanaceae* and *Chenopodiaceae* families [1]. The soil-borne protist, *Spongospora subterranean*, causal agent of potato powdery scab, is a vector of PMTV [2]. PMTV infection mainly manifests in tubers as internal necrosis and results in reduced yield and quality [3]. Since its first diagnosis in UK in 1966 [4], PMTV has been identified in most potato cultivation areas across Asia, Europe, North and South America [5–8]. Phylogenetic evidence supports the hypothesis that PMTV originated from the Andean region of South America [7, 8]. Managing PMTV continues to be a challenge [9]. There are very few resistant cultivars available [3]. The extremely long viability (up to 18 years) of PMTV in its vector’s dormant spores further increases the difficulty for disease management via crop rotation [10]. Thus, the best practice for PMTV control is early-stage diagnosis to prevent its spread.

PMTV genome comprises of three single-stranded (ss) (+) RNAs [11–13]. The three RNA fragments share highly conserved 5’-untranslated regions and an identical tRNA-like structure in their 3’-end [14]. The genomic RNAs encode multiple proteins that are essential for virus replication and propagation. The PMTV RNA-dependent RNA polymerase (RdRp), which is responsible for viral genome replication, is encoded by the longest 6043-nt Rep RNA [13]. The coat protein (CP) and another larger structural protein Coat Protein Read Through (CPRT) are both translated from the intermediate-length (3134 nt) CP RNA, with CPRT being translated via read through of a stop codon [12]. The triple gene block (TGB) RNA is 2964 nucleotides (nt) in length, which encodes a cysteine-rich RNA-silencing suppressor and three movement proteins (TGB1–3) with overlapping open reading frames (ORFs). Both CPRT [15] and the three TGBs [16, 17] facilitate the movement of PMTV particles across plant cells, with the physical interaction between CPRT and TGB1 being critical for this systemic movement process [18].

One of the most effective means to reduce PMTV infection or introduction into a virus-free field is by ensuring that the seed tubers are free of PMTV infection. Several molecular detection methods have been reported for PMTV. One of the earlier reports using reverse transcription quantitative PCR (RT-qPCR) approach [19] has now been digitalized for direct detection of PMTV in soil samples [20]. Many of the protocols require laboratory-based sample processing followed by RT-PCR or RT-qPCR methods that require 4 to 8 hours to obtain the result. In situations where the virus status of a particular field of potatoes or shipments is urgently needed, a rapid test that can be carried out in the field or on-site such as shipping point inspections would greatly aid in seed certification programs, trade, and movement of produce across state or country lines. Recombinase polymerase amplification (RPA), first described in 2006 [21], is gaining wide adaptation due to its convenience, faster processing, high specificity, and sensitivity [22]. In this study, we developed a rapid, RPA-based on-site test for PMTV detection in potato leaves and tubers. The method combines the convenience of the RPA assay with a simple sample extraction procedure, and provides diagnostic limit of detection (LOQ) similar to that of traditional molecular methods.

## Materials and methods

The protocol described in this peer-reviewed article is published on protocols.io (http://dx.doi.org/10.17504/protocols.io.14egn7n7qv5d/v1), and is included for printing as S1 File with this article.

### Plant materials

Potato tubers with suggestive of PMTV-induced symptoms were collected from experimental plots in Idaho and Washington, USA, during 2019 and 2020 growth seasons.

### Design of RPA primers and probes

Sequences of PMTV RNA 1, 2, and 3 segments were obtained from GenBank (https://www.ncbi.nlm.nih.gov/genbank/). Their conserved homologous regions were used to design primers and probes following RPA design guidelines outlined in the help book of Agdia AmplifyRP^TM^ XRT Discovery Kit (https://orders.agdia.com/pathogen-tests/amplifyrp/amplifyrp-xrt-discovery). Oligos were screened *in silico* for efficacy analyses of melting temperature, potential secondary structure, heterodimer activity between oligos, and potential cross reactivity to other pathogens. Likewise, highly conserved plant gene sequences were aligned and used for RPA oligo design to serve as an endogenous control. Primers and probes were synthesized by Integrated DNA Technologies (IDT, Coralville, IA, USA). PMTV and internal control probes were labeled with a 6-carboxyfluorescein (FAM) fluorescent dye and ZEN^TM^ internal quencher, and a carboxy-X-rhodamine (ROX) fluorescent dye and TAO^TM^ internal quencher, respectively (Table 1).

**Table 1:** PCR primers used for comparison analysis in the RPA assay for the detection of potato mop-top virus.

Primer	Sequence (5’-3’)	Target	Position	Amplicon (bp)	Source
PMTV-2/3F	AGAGCAGCCGTCGAGAATAG	RNA3	338	416	[25]
PMTV-2/3R	TCGTCCACCTCTGCGAGTTG		753		
PMTV-D-F	AGAATTGRCATCGAAACAGCA	RNA 2	1710	69	[24]
PMTV-D-R	GTCGCGCTCCAATTTCGTT		1778		
PMTV-D-P*	CCACAAACAGACAGGTATGGTCCGGAA		1741		
PMTV-1948F	GTGATCAGATCCGCGTCCTT	RNA 3	1948	70	[19]
PMTV-2017R	CCACTGCAAAAGAACCGATTTC		2017		
PMTV-1970*	ACCAGAACTACGGTGCCGCGTCG		1970		

*Probes labeled with FAM at 5’-end and Black Hole Quencher (BHQ) 1 at 3’-end.

### Sample preparation

Potato leaves, petioles, tubers and tissue culture plantlets were used for extraction. Foliar tissues were extracted in a mesh bag (Agdia, ACC 00930) using general extraction buffer (Agdia, ACC 00955) with a weight to volume ratio of 1:10 (1gr:10mL). Four cores per tuber were taken for test using a biopsy tool (Harris Uni-Core 3mm, Sigma WHAWB100039). Cores from up to ten tubers were combined into a single sample and extracted in a mesh bag using general extraction buffer with a 1:2 weight to buffer ratio. Samples were homogenized with a blunt object and left for five minutes at room temperature. Extracted sap was used for direct PMTV detection with RPA reaction pellets (Agdia Inc., Elkhart, IN, USA, Catalog number XCS 99200).

### RNA extraction

RNA extraction was performed as described previously [23]. Two grams of fresh tissue was ground in liquid nitrogen using mortar and pestle. The fine powder was then transferred to a 15 mL Falcon tube and the following were sequentially: 1.0 mL of 5 M NaCl, 0.5 mL of 10% (w/v) SDS, 1.65 mL of 1.95% (w/v) Na_2_SO_3_, 1.75 mL of 0.2 M borate-Tris buffer (pH 8.0 with 10 mM EDTA), and 0.1 mL of β-mercaptoethanol. After mixing by a vortexer, the mixture was incubated at 65°C for 5 min. The supernatant, obtained by centrifugation at 1800 g for 5 min at 23°C, was mixed with an equal volume of Tris-saturated phenol (pH 7.9). After centrifugation, the upper phase was extracted with an equal volume of chloroform-isoamyl alcohol (24:1) and centrifuged again to facilitate phase separation. The extract was mixed with isopropanol at the ratio of 1: 0.9. RNA was pelleted by centrifuging at 20,000 g (15 min, 4°C) after incubation at 4°C for 1 h. After washing with 1 mL 70% ethanol for four times, the pellet was dissolved in RNase-free water.

### RPA amplification and detection

RNA (one or two microliters) was added directly to the reaction tube containing reaction primers, probes and Discovery XRT pellets rehydrated with 25 μL of kit reagents (Agdia, XCS 99200), ensuring that the pellets were completely dissolved. Rehydrated reactions were then placed into a pre-warmed AmpliFire^TM^ isothermal fluorometer (Agdia, AFR 60400), set at 42°C and monitored for 20 min on the FAM and ROX/CalRed channels. After every 4 min of incubation, the reaction tubes were removed from the AmpliFire, mixed by tapping with fingers, briefly centrifuged, and were placed back in the AmpliFire (S1 File).

### RT-qPCR

Two one-step RT-qPCR assays (A&B) were carried out to compare the results with those from the RPA. The first assay [24] was developed to target RNA 2 of the type isolate (GenBank no. NC_003725) and a portable real-time PCR machine was used. In our assay, the reaction was run on the stationary BioRad CFX96 as a one-step 25μL RT-qPCR reaction utilizing that consisted of 1x TaqMan GTXpress reaction master mix (Thermo Fisher, 4401892), 0.2 μM PMTV-D-F and R primers, 0.15 μM PMTV-D-P labeled with FAM and BHQ, 50 U M-MLV reverse transcriptase (Promega, M1705), and 2 μL of purified RNA (1pg/uL, 100fg/ μL, 100fg/ μL and 100fg/ μL). The second RT-qPCR assay [19] was developed to target RNA 3 of the type isolate (GenBank no. NC_003724). This set was run as an identical one-step 25 μL RT-qPCR reaction except using 0.3 μM PMTV-1948F and PMTV-2017R primers. Reactions for the RNA 2 target were run at 50°C for 30 min, 95°C for 10 min, 50 cycles at 95° C for 15 sec and 60°C for 1 min. Reactions for the RNA3 target used an initial RT at 48°C for 15 minutes and only 40 cycles. Data were analyzed using the BioRad CFX Manager ^TM^ to set the threshold of relevant positive and negative controls.

RPA results were compared with those obtained from conventional two-step RT-PCR. The RT-PCR assay for PMTV detection [25] was developed to produce a 416-bp amplicon from PMTV RNA 3 encoding CPRT. Reactions were run according to previously described specifications. In brief, reverse transcription was carried out using 0.5 μM PMTV2/3R, 1x PCR reaction buffer, 5 mM MgCl_2_, 1 mM dNTPs, 20 U RNase inhibitor (Promega, N2611), 50 U M-MLV and 2 μL of purified RNA (1pg/μL, 100fg/ μL, 100fg/ μL and 100fg/ μL) in a 20 μL volume. RNA was denatured for 5 min at 75°C with the reverse primer and water prior to running RT. The RT reaction was performed at 42°C for 30 min followed by 99°C for 5 min. The subsequent PCR reaction was set up as following: 1x PCR buffer, 1.5 mM MgCl_2_, 0.1 mM dNTPs, 0.4 μM PMTV 2/3 F and R, 1.25 U HotStar Taq polymerase (Qiagen, 203205), and 2 μL of cDNA in a 25μL volume. The cycling profile was 95°C for 15 min, followed by 30 cycles of 94°C for 15 sec, 58°C for 1 min and 72°C for 30 sec, and a final extension at 72°C for 7 min. DNA bands were separated on a 1.5% agarose gel and stained with ethidium bromide.

### Sensitivity analysis

A chimeric DNA fragment was designed based on the TGB1 [24] and coat protein [19]-encoding genes from the PMTV type isolate sequence (GenBank no. NC_003725 & NC_003724) by combining the amplicon sequences for RT-RPA, RT-qPCR, and RT-PCR with a T7 RNA polymerase tag. The chimeric DNA was synthesized by Integrated DNA Technologies (IDT, Coralville, IA, USA). RNA transcripts were produced *in vitro* via T7 RNA transcription following manufacturer’s recommendations for non-labeled RNA synthesis (Promega, P2075). RNA concentration and purity were measured with a Thermo Fisher NanoDrop-2000 Spectrophotometer, and samples were stored at -80°C after adding RNasin Plus RNase inhibitor (Promega, N2615).

## Results and discussion

### Screening of oligonucleotide combinations for PMTV detection

**Fig 1:**
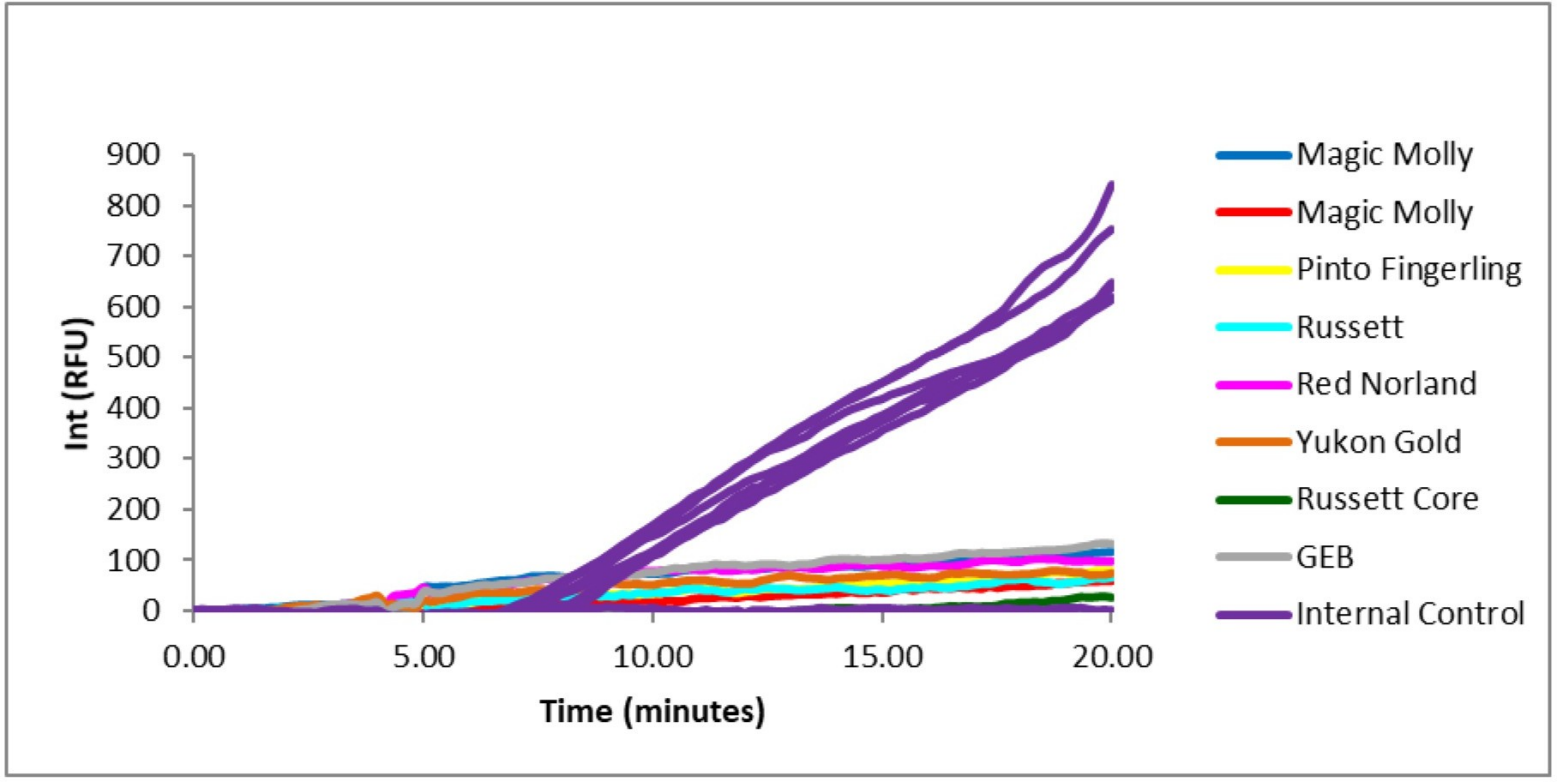
Healthy potato tissues produced amplification of the internal control but not potato mop-top virus (PMTV) when using TGB1 combination P1F1R3. Internal control values were consistent across five varieties of leaf tissues and one variety of potato tubers. General extraction buffer did not result in erroneous results of the designed assay.

Eighteen out of a total of 22 oligonucleotide combinations that were screened successfully detected PMTV in a naturally infected sample (Table 2). Combinations targeting the TGB1 yielded the strongest amplification (S1 Fig). These combinations paired with the internal control amplified the target region from three additional PMTV-infected samples. TGB1-P1F1R2, however, resulted in false positives with healthy controls. TGB1-P1F1R3 could not be amplified from healthy samples, while the internal control was amplified from healthy samples, which suggests no false positives from healthy cultivars (Fig 1). An oligo combination TGB1-P1F1R3 was used for further RPA tests.

**Table 2:** RPA XRT screening of oligos for potato mop-top virus (PMTV) detection using RNA from a tuber. Sample onset time and intensity are provided as averages for two replicates of each combination. Sample onset time was determined by evaluating change in fluorescence intensity over time, set arbitrarily at 60 RFU/min. Intensity is an evaluation of the fluorescence output of a given oligo combination. Combinations that failed to amplify PMTV resulted in a “-” for no onset and intensities below 200 mV.

Target	Probe	Forward	Reverse	Onset (min)	Intensity (mV)
CPRT	P1	F1	R1	10.00	1883
CPRT	P1	F1	R2	16.33	538
CPRT	P1	F2	R1	9.00	2089
CPRT	P1	F2	R2	15.33	780
CPRT	P1	F3	R1	7.33	1997
CPRT	P1	F3	R2	13.3	1287
TGB1	P1	F1	R1	7.33	2542
TGB1	P1	F1	R2	6.00	4365
TGB1	P1	F1	R3	7.00	3840
RNA1	P1	F1	R1	11.33	1779
RNA1	P1	F1	R2	-	145
RNA1	P1	F2	R1	14.00	815
RNA1	P1	F2	R2	-	58
RNA1	P1	F3	R1	14.00	975
RNA1	P1	F3	R2	-	40
RNA1	P2	F4	R3	11.67	2167
RNA1	P2	F4	R4	19.33	323
RNA1	P2	F4	R5	18.00	575
RNA1	P2	F5	R3	13.67	1385
RNA1	P2	F5	R4	11.67	2594
RNA1	P2	F5	R5	18.00	391
RNA1	P2	F6	R4	-	235
RNA1	P2	F6	R6	-	202

### The PMTV RPA assay is highly specific

To confirm the high specificity of the assay, closely related potato viruses and PMTV isolates from diverse locations were tested. A list of tested potato viruses and PMTV isolates and their reaction results in the RPA assay are presented in Table 3. None of the potato viruses other than PMTV were detected by the RPA assay, suggesting no potential cross reactions. For each assay, the internal control was amplified, as expected, from both PMTV negative and positive samples. PMTV isolates from different sources could be detected.

**Table 3:** List of viruses tested to determine the specificity of the RPA assay. The multiplexed internal control amplifies the 18s rNRA of host plants and ensures the suitability of the potato mop-top virus (PMTV) results from a variety of host tissues, indicating successful reactions and true negative PMTV results.

			Results	
Pathogen	Source	Host	PTMV	Internal Control
*Beet Soil-borne virus*	DSMZ	*Beta vulgaris*	-,-	+,+
*Potato virus A*	Agdia, Inc	*Nicotiana benthamiana*	-,-	+,+
*Potato latent virus*	Agdia, Inc.	*Solanum tuberosum*	-,-	+,+
*Potato virus S*	Agdia, Inc.	*Solanum tuberosum*	-,-	+,+
*Potato virus X*	Agdia, Inc.	*Solanum tuberosum*	-,-	+,+
*Potato acuba mosaic virus*	Agdia, Inc	*Nicotiana rustica*	-,-	+,+
*Potato virus Y*	Agdia, Inc.	*Solanum tuberosum*	-,-	+,+
*Tomato spotted wilt orthotospovirus*	Agdia, Inc.	*Capsicum annuum*	-,-	+,+
*Tobacco rattle virus*	ATCC	*Nicotiana tabacum*	-,-	+,+
*Potato virus T*	DSMZ	*Nicotiana hesperis*	-,-	+,+
*Tobacco etch virus*	DSMZ	*Nicotiana tabacum*	-,-	+,+
*Potato virus V*	DSMZ	*Nicotiana benthamiana*	-,-	+,+
*Potato virus M*	DSMZ	*Nicotiana occidentalis*	-,-	+,+
*Potato leafroll virus*	DSMZ	*Physalis floridana*	-,-	+,+
*Alfalfa mosaic virus*	DSMZ	*Nicotiana rustica*	-,-	+,+
*Andean potato mottle virus*	DSMZ	*Nicotiana glutinosa*	-,-	+,+
*Potato mop-top virus*	Montana State University	*Solanum tuberosum*	+,+	+,+
*Potato mop-top virus*	Montana State University	*Solanum tuberosum*	+.+	+,+
*Potato mop-top virus*	DSMZ	*Solanum tuberosum*	+,+	+,+
*Potato mop-top virus*	Bioreba	*Solanum tuberosum*	+,+	+,+

### The PMTV RPA and RT-qPCR assays has similar LOQs

**Fig 2:**
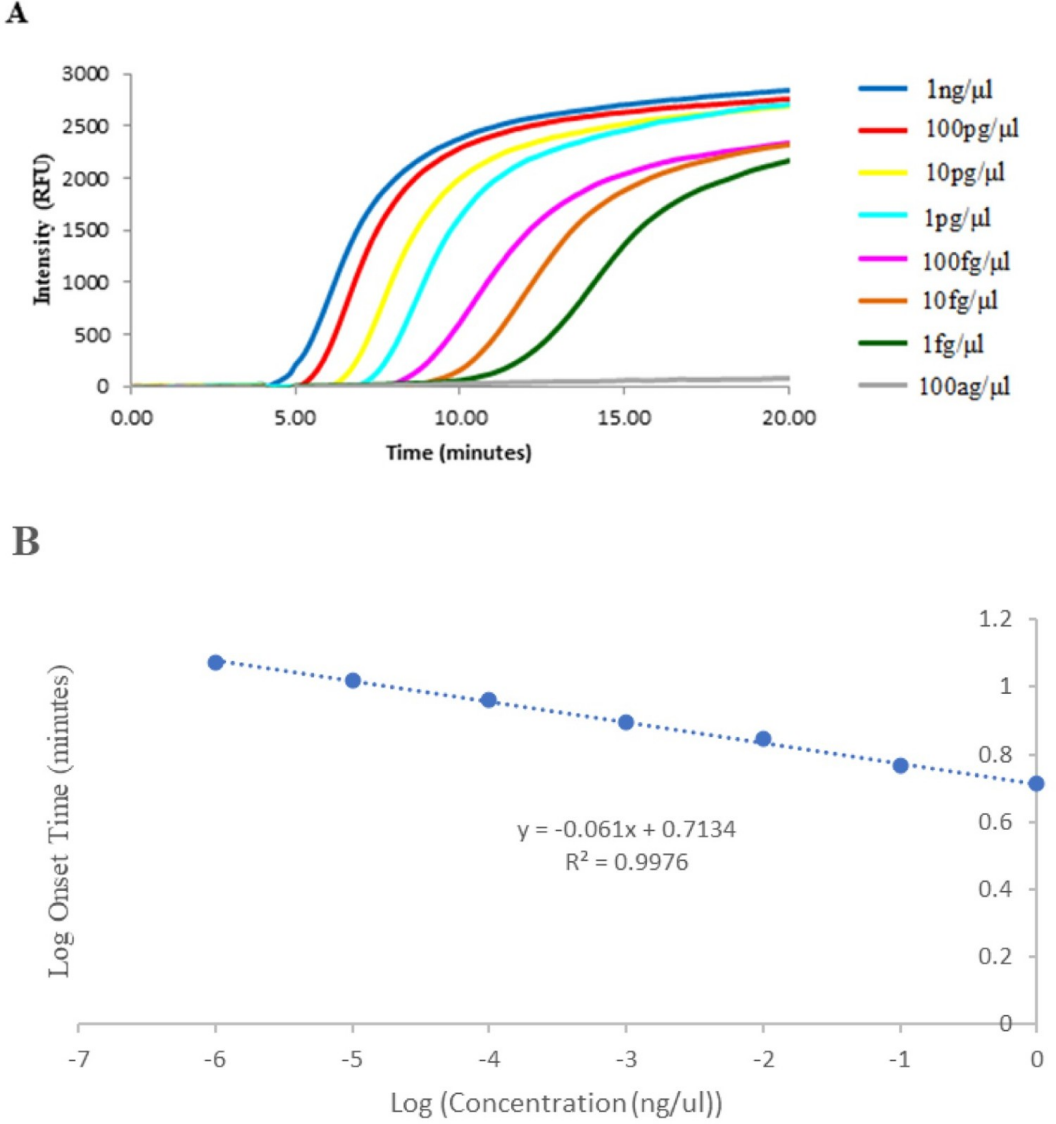
Detection limits of the RPA assay for potato mop-top virus (PMTV). (A) Serial dilution of PMTV RNA in water exhibited uniform amplification and detection from 1 ng/μL to 1 fg/μL. (B) Logarithmic comparison of the onset time to the concentration of RNA template generated a linear prediction curve, allowing for a quantitative analysis of purified RNA template.

The PMTV RPA assay could detect as little as 10 fg/μL of RNA transcript using a multitude of potato tissues and cultivars as the crude extract diluents (Table 4, Fig 2). At times, the detection threshold could be down to 1 fg/μL, but this was not consistently achieved from all tested tissues, particularly tuber extracts. Detection at 10 fg/uL has also been attained from multiple preparations of the RNA transcript defining the limit of detection (LOQ). The LOQ of our RPA assay was comparable to that of RT-qPCR and lower than that of the conventional RT-PCR by ten folds.

**Table 4:** Comparative sensitivity analysis between RPA and other published molecular methods for the detection of potato mop-top virus (PMTV). PMTV RNA transcript was diluted and tested in duplicate by following the appropriate protocol for each method. Cq data is provided for the RT-qPCR assays and “+” and “-” indicate samples are positive and negative of PMTV for qualitative assays.

Diluent	RT-qPCR method A [24]	RT-qPCR method B [19]	RT-PCR [25]	RPA			
				Water	Leaf	Tuber	Sprout
	Water	Water	Water				
1pg**/** μL	**25.17, 24.99**	**27.95, 27.75**	**+, +**	**+, +**	**+, +**	**+, +**	**+, +**
100fg**/** μL	**28.81, 28.73**	**32.87, 32.70**	**-, -**	**+, +**	**+, +**	**+, +**	**+, +**
10fg**/** μL	**31.82, 32.25**	**35.58, 35.27**	**-, -**	**+, +**	**+, +**	**+, +**	**+, +**
1fg**/** μL	**34.66, 34.19**	**37.94, 37.74**	**-, -**	**+, +**	**+, +**	**-, -**	**+, +**

### PMTV detection using real-time duplex RPA assay

**Fig 3:**
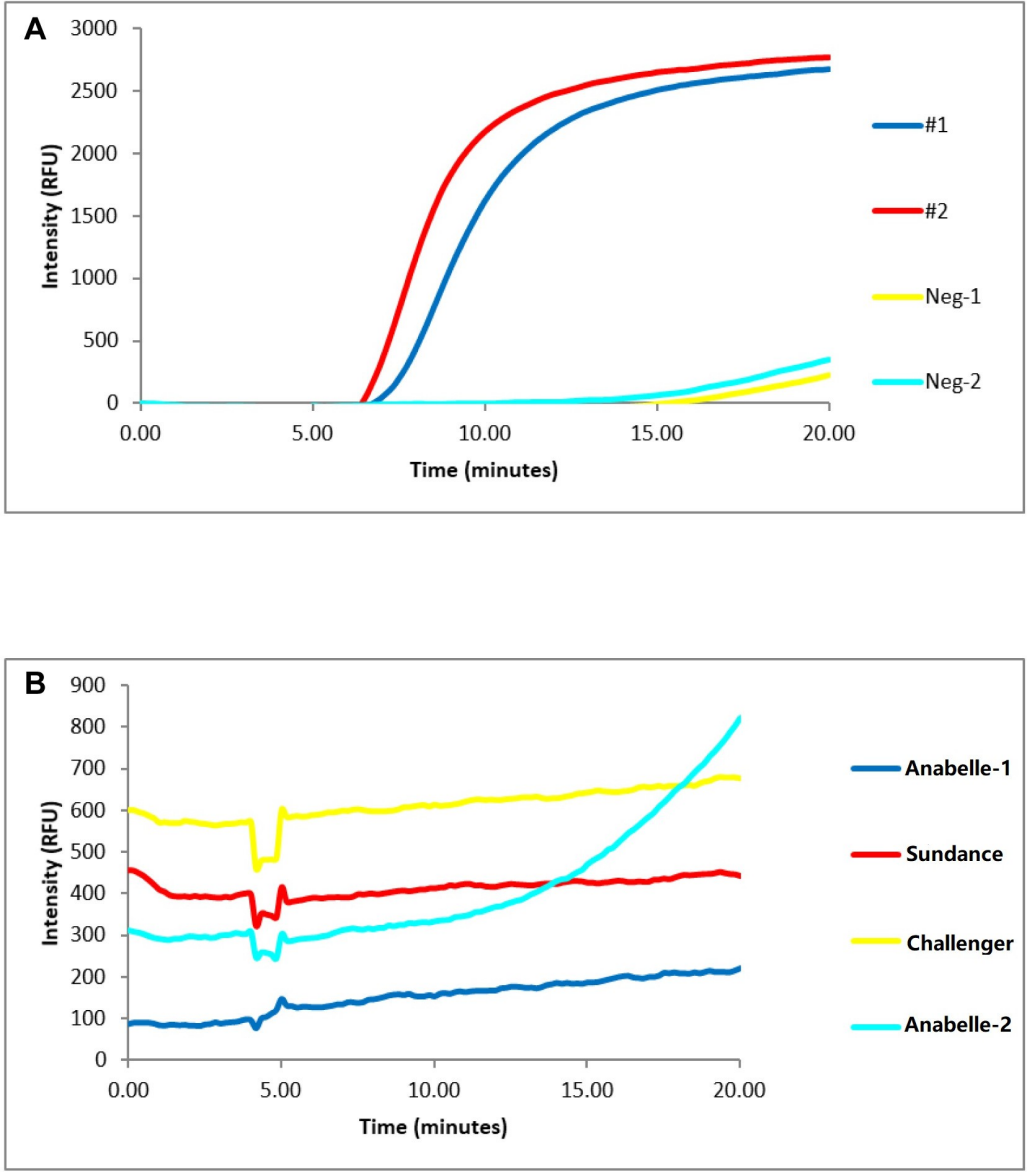
Detection of potato mop-top virus (PMTV) using real-time duplex RPA assay. (A) RNA samples from two infected tubers and two healthy tubers. (B) Tuber samples were prepared by crude extraction.

RNA samples from two tubers with virus-like symptoms were tested in parallel with two negative controls. Both RNA samples from the suspect tubers showed amplification of the PMTV fragment. The negative controls exhibited no PMTV amplification as expected (Fig 3A). In addition, crude sap extracts from four potato tubers collected in North Dakota, USA were tested by PMTV-RPA. Sample Annabelle-2, which showed typical PMTV-infection symptoms, gave positive amplification of PMTV fragment (Fig 3B). These results further validated the efficacy of our PMTV-RPA assay.

## Conclusion

Results presented here described and demonstrated the applicability of RPA for potential on-site PMTV detection. The test includes an internal control to assess reaction performance in the presence of difficult-to-handle tissues and/or cultivar variation. The assay utilizes a crude general extraction buffer and maceration to process the sample. Reactions were performed at an isothermal temperature for 20 min to increase sample throughput. The assay is capable of detecting all PMTV isolates tested, as confirmed by PCR. *In silico* data support successful detection of all currently described PMTV-genetic variants (S1 Table) with no cross reaction to closely related viruses or other potato-infecting pathogens. The LOQ of our RPA assay is comparable to those of existing diagnostic methods. The cost of traditional RT-PCR diagnosis, including RNA extraction, reverse transcription, PCR primers and reagents, is expected to be more than $20 per sample, in addition to the time devoted to processing the samples and data interpretation. In contrast, this assay only needs crude sample extraction and the PMTV kit ($13.65 per reaction) run on either a qPCR machine or a fluorometer for 20 minutes. In conclusion, the RPA protocol described here presents a low-cost, sensitive, rapid and reliable tool for onsite monitoring of PMTV infection status in potato production chain.

## Supporting information

S1 Fig RPA primer and probe combinations from Table 2 screened against naturally infected PMTV infected tubers. All primers were screened at 0.4 μM and probes at 0.08 μM. Results for each target region are displayed in A) Coat protein read through protein, CPRT; B) Triple gene block protein 1, TGB1; C) RNA 1 probe 1 combination; D) RNA 1 probe 2 combinations. Combinations targeting the TGB1 gene had the strongest amplification curves and were used for further testing and refinement.
(DOCX)

S1 Table Assessment of the PMTV RPA oligo combination TGB1-P1F1R3 to sequences in the NCBI GenBank database to determine in silico feasibility of detection. The length of each oligo is present below the identifier with the corresponding number of matching nucleotides described for each PMTV isolate under each oligonucleotide.
(DOCX)

S1 File Rapid RNA amplification test for PMTV. Also available on protocols.io. http://dx.doi.org/10.17504/protocols.io.14egn7n7qv5d/v1.
(PDF)

## References

[1] Kirk HG. *Mop-top virus*, relationship to its vector. Am. J. Potato Res. 2008; 85:261–265. 10.1007/s12230-008-9021-7

[2] Gau RD, Merz U, Falloon RE, Brunner PC. Global genetics and invasion history of the potato powdery scab pathogen, *Spongospora subterranea f*.*sp*. *subterranea*. PLoS One. 2013; 8:e67944. doi: 10.1371/journal.pone.0067944 23840791 PMC3695870

[3] Yellareddygari SKR, Whitworth JL, Gudmestad NC. Assessing potato cultivar sensitivity to tuber necrosis caused by *Potato mop-top virus*. Plant Dis. 2018; 102:1148–1153. doi: 10.1094/PDIS-10-17-1585-RE 30673438

[4] Calvert EL, Harrison BD. *Potato mop-top*, a soil borne virus. Plant Pathol. 1966; 15:31–40. 10.1111/j.1365-3059.1966.tb00333.x

[5] Ramesh SV, Raikhy G, Brown CR, Whitworth JL, Pappu HR. Complete genomic characterization of a *Potato mop-top virus* isolate from the United States. Arch. Virol. 2014; 159:3427–3433. doi: 10.1007/s00705-014-2214-0 25287129

[6] Beuch U, Berlin S, Åkerblom J, Nicolaisen M, Nielsen SL, Crosslin JM, et al. Diversity and evolution of *Potato mop-top virus*. Arch. Virol. 2015; 160:1345–1351. doi: 10.1007/s00705-015-2381-7 25753427

[7] Kalyandurg P, Gil JF, Lukhovitskaya NI, Flores B, Müller G, Chuquillanqui C, et al. Molecular and pathobiological characterization of 61 *Potato mop-top virus* full-length cDNAs reveals great variability of the virus in the centre of potato domestication, novel genotypes and evidence for recombination. Mol. Plant Pathol. 2017; 18:864–877. doi: 10.1111/mpp.12552 28390168 PMC6638219

[8] Zhai Y, Mallik I, Hamid A, Tabassum A, Gudmestad N, Gray S, et al. Genetic diversity in *Potato mop-top virus* populations in the United States and a global analysis of the PMTV genome. Eur. J. Plant Pathol. 2020; 156:333–342. 10.1007/s10658-019-01836-6

[9] Domfeh O, Bittara FG, Gudmestad NC. Sensitivity of potato cultivars to *Potato mop-top virus*-induced tuber necrosis. Plant Dis. 2015; 99:788–796. doi: 10.1094/PDIS-07-14-0705-RE 30699525

[10] Santala J, Samuilova O, Hannukkala A, Latvala S, Kortemaa H, Beuch U, et al. Detection, distribution and control of *Potato mop-top virus*, a soil-borne virus, in northern Europe. Ann. Appl. Biol. 2010; 157:163–178. 10.1111/j.1744-7348.2010.00423.x

[11] Scott KP, Kashiwazaki S, Reavy B, Harrison BD. The nucleotide sequence of *Potato mop-top virus* RNA 2: A novel type of genome organization for a *Furovirus*. J. Gen. Virol. 1994; 75:3561–3568. doi: 10.1099/0022-1317-75-12-3561 7996148

[12] Kashiwazaki S, Scott KP, Reavy B, Harrison BD. Sequence analysis and gene content of *Potato mop-top virus* RNA 3: further evidence of heterogeneityin the genome organization of *Furoviruses*. Virology. 1995; 206:701–706. doi: 10.1016/s0042-6822(95)80092-1 7831829

[13] Savenkov EI, Sandgren M, Valkonen JP. Complete sequence of RNA 1 and the presence of tRNA-like structures in all RNAs of *Potato mop-top virus*, genus *Pomovirus*. J. Gen. Virol. 1999; 80:2779–2784. doi: 10.1099/0022-1317-80-10-2779 10573175

[14] Gil JF, Adams I, Boonham N, Nielsen SL, Nicolaisen M. Molecular and biological characterization of *Potato mop-top virus* (PMTV, *Pomovirus*) isolates from potato growing regions in Colombia. Plant Pathol. 2016; 65:1210–1220. 10.1111/ppa.12491

[15] Reavy B, Arif M, Cowan GH, Torrance L. Association of sequences in the coat protein/readthrough domain of *Potato mop-top virus* with transmission by S*pongospora subterranea*. J. Gen. Virol. 1998; 79:2343–2347. doi: 10.1099/0022-1317-79-10-2343 9780038

[16] Samuilova O, Santala J, Valkonen JPT. Tyrosine phosphorylation of the triple gene block protein 3 regulates cell-to-cell movement and protein interactions of *Potato mop-top virus*. J. Virol. 2013; 87:4313–4321 doi: 10.1128/JVI.03388-12 23365450 PMC3624400

[17] Lukhovitskaya NI, Cowan GH, Vetukuri RR, Tilsner J, Torrance L, Savenkov EI. Importin-α-mediated nucleolar localization of *Potato mop-top virus* TRIPLE GENE BLOCK1 (TGB1) protein facilitates virus systemic movement, whereas TGB1 self-interaction is required for cell-to-cell movement in *Nicotiana benthamiana*. Plant Physiol. 2015; 167:738–752. doi: 10.1104/pp.114.254938 25576325 PMC4348779

[18] Torrance L, Lukhovitskaya NI, Schepetilnikov MV, Cowan GH, Ziegler A, Savenkov EI. Unusual long-distance movement strategies of Potato mop-top virus RNAs in *Nicotiana benthamiana*. Mol. Plant–Microbe Interact. 2009; 22:381–390. doi: 10.1094/MPMI-22-4-0381 19271953

[19] Mumford RA, Walsh K, Barker I, Boonham N. Detection of *Potato mop top virus* and *Tobacco rattle virus* using a multiplex real-time fluorescent reverse-transcription polymerase chain reaction assay. Phytopathology. 2000; 90:448–453. doi: 10.1094/PHYTO.2000.90.5.448 18944548

[20] Pandey B, Mallik I, Gudmestad NC. Development and application of a real-time reverse-transcription PCR and droplet digital PCR assays for the direct detection of *Potato mop top virus* in soil. Phytopathology. 2020; 110:58–67. doi: 10.1094/PHYTO-05-19-0185-FI 31448996

[21] Piepenburg O, Williams CH, Stemple DL, Armes NA. DNA detection using recombination proteins. PLoS Biol. 2006; 4: e204. doi: 10.1371/journal.pbio.0040204 16756388 PMC1475771

[22] Wang XF, Xie SL, Chen XY, Peng C, Xu XL, Wei W, et al. A rapid and convenient method for on-site detection of MON863 maize through real-time fluorescence recombinase polymerase amplification. Food Chem. 2020; 15:126821. doi: 10.1016/j.foodchem.2020.126821 32361093

[23] Kumar GN, Iyer S, Knowles NR. Extraction of RNA from fresh, frozen, and lyophilized tuber and root tissues. J Agric Food Chem. 2007; 55:1674–1678. doi: 10.1021/jf062941m 17288445

[24] DeShields JB, Bomberger RA, Woodhall JW, Wheeler DL, Moroz N, Johnson DA, et al. On-site molecular detection of soil-borne phytopathogens using a portable real-time PCR system. J. Vis. Exp. 2018; 132:56891. doi: 10.3791/56891 29553557 PMC5931365

[25] Crosslin JM, Hamlin L. Standardized RT-PCR conditions for detection and identification of eleven viruses of potato and potato spindle tuber viroid. Am. J. Potato Res. 2011; 88:333–338. 10.1007/s12230-011-9198-z

