# Supplementary material for: An on-site adaptable test for rapid and sensitive detection of Potato mop-top virus, a soil-borne virus of potato (Solanum tuberosum)
Source: PLoS One. 2022 Aug 1;17(8):e0270918. doi: 10.1371/journal.pone.0270918 (PMC9343021; doi:10.1371/journal.pone.0270918)
Supplement: S1 Fig — All primers were screened at 0.4 μM and probes at 0.08 μM. Results for each target region are displayed in A) Coat protein read through protein, CPRT; B) Triple gene block protein 1, TGB1; C) RNA 1 probe 1 combination; D) RNA 1 probe 2 combinations. Combinations targeting the TGB1 gene had the strongest amplification curves and were used for further testing and refinement. (DOCX) [file pone.0270918.s001.docx]

A

B


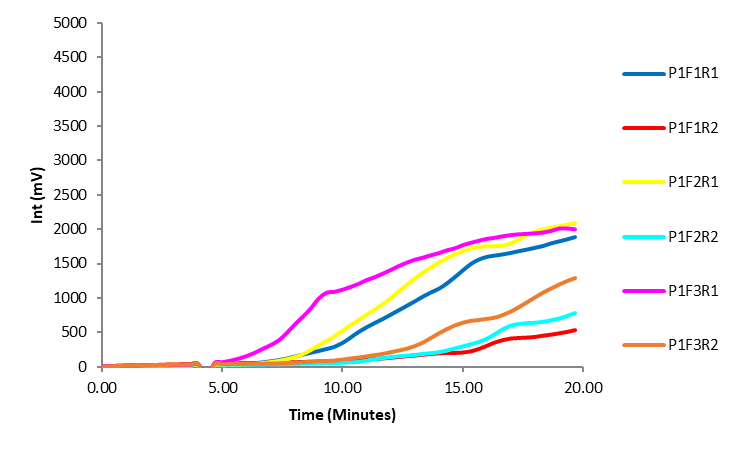

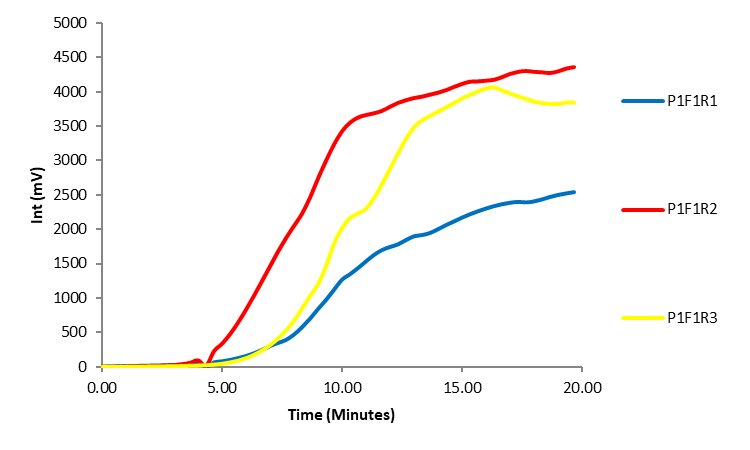


C

D


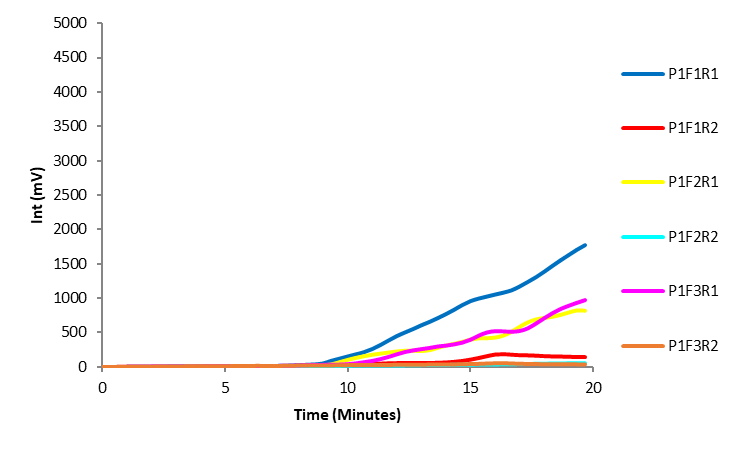

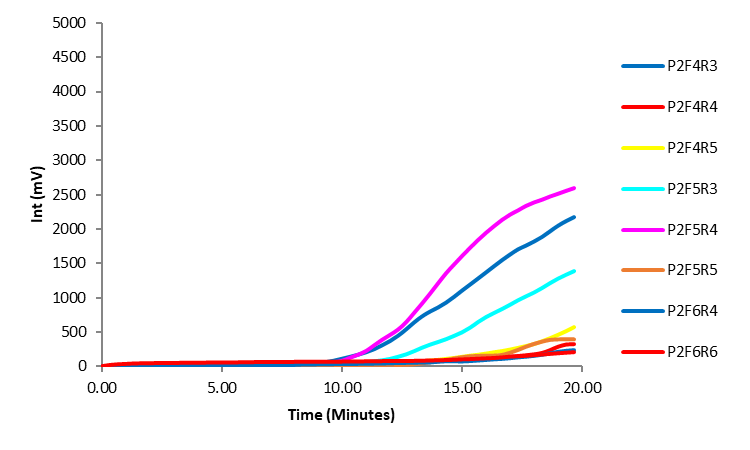


**S1 Fig.** RPA primer and probe combinations from Table 2 screened against naturally infected PMTV infected tubers. All primers were screened at 0.4 μM and probes at 0.08 μM. Results for each target region are displayed in A) Coat protein read through protein, CPRT; B) Triple gene block protein 1, TGB1; C) RNA 1 probe 1 combination; D) RNA 1 probe 2 combinations. Combinations targeting the TGB1 gene had the strongest amplification curves and were used for further testing and refinement.
