# Supplementary material for: An on-site adaptable test for rapid and sensitive detection of Potato mop-top virus, a soil-borne virus of potato (Solanum tuberosum)
Source: PLoS One. 2022 Aug 1;17(8):e0270918. doi: 10.1371/journal.pone.0270918 (PMC9343021; doi:10.1371/journal.pone.0270918)
Supplement: S1 Table — The length of each oligo is present below the identifier with the corresponding number of matching nucleotides described for each PMTV isolate under each oligonucleotide. (DOCX) [file pone.0270918.s002.docx]

**S1 Table.** Assessment of the PMTV RPA oligo combination TGB1-P1F1R3 to sequences in the NCBI GenBank database to determine *in silico* feasibility of detection. The length of each oligo is present below the identifier with the corresponding number of matching nucleotides described for each PMTV isolate under each oligonucleotide.

| **GenBank Accession Number** | **TGB1-F1 (35bp)** | **TGB1-R3 (31bp)** | **TGB1-P1 (50bp)** | **Predicted Detection** |
| --- | --- | --- | --- | --- |
| AJ277556 | 35 | 29 | 50 | YES |
| AY187010 | 35 | 31 | 50 | YES |
| AY353719 | 35 | 31 | 50 | YES |
| AY426745 | 35 | 31 | 50 | YES |
| D30753 | 35 | 29 | 50 | YES |
| DQ144451 | 35 | 31 | 50 | YES |
| JX889608 | 35 | 30 | 50 | YES |
| JX889609 | 35 | 29 | 50 | YES |
| KM822704 | 35 | 31 | 50 | YES |
| KM822705 | 35 | 29 | 50 | YES |
| KM822706 | 35 | 31 | 50 | YES |
| KM822707 | 35 | 31 | 50 | YES |
| KM822708 | 35 | 31 | 50 | YES |
| KP420028 | 35 | 31 | 50 | YES |
| KR857351 | 35 | 29 | 50 | YES |
| KR857354 | 35 | 29 | 50 | YES |
| KR857357 | 35 | 31 | 50 | YES |
| KR857360 | 35 | 31 | 50 | YES |
| KR857363 | 34 | 29 | 50 | YES |
| KU955496 | 35 | 30 | 50 | YES |
| KU955497 | 35 | 30 | 50 | YES |
| KU955498 | 34 | 30 | 50 | YES |
