# Supplementary material for: An on-site adaptable test for rapid and sensitive detection of Potato mop-top virus, a soil-borne virus of potato (Solanum tuberosum)
Source: PLoS One. 2022 Aug 1;17(8):e0270918. doi: 10.1371/journal.pone.0270918 (PMC9343021; doi:10.1371/journal.pone.0270918)
Supplement: S1 File — Also available on protocols.io. http://dx.doi.org/10.17504/protocols.io.14egn7n7qv5d/v1. (PDF) [file pone.0270918.s003.pdf]

dx.doi.org/10.17504/protocols.io.14egn7n7qv5d/v1

An on-site adaptable test for rapid and sensitive detection of *Potato mop-top virus*, a soil-borne virus of potato (*Solanum tuberosum*)

Rapid RNA Amplification Test for PMTV

1. Cut core tubers with blade. Take 3 - 4 cores per tuber.

2. Extract with GEB extraction buffer at a ratio of 1:2 (w:v) in a mesh extraction bag (0.3 g/3 mL). Let rest for 5 minutes at room temperature.

3. Remove one colored PD1 filled tube for each sample being tested. Individual tubes may be cut from the strip of tubes using scissors.

4. Transfer 5 µL of sample extract into the tube containing PD1 diluent and mix well.

5. Press the "Execute Reaction" button on the AmpliFire®. Then Scan PMTV Product Code.

6. Remove a canister of reaction pellets from the white foil pouch labeled with the barcode. Then remove a strip of reaction pellets from the desiccated container.

Note: Reaction Pellets are light sensitive. Immediately place remaining reaction pellets back into the desiccated tube and then insert the desiccant tube into the foil pouch to protect from light.

7. Transfer 25 µL from the colored tube from step 4, into the reaction pellet (clear tube). Mix well and spin down.

8. Press "Start" on the AmpliFire. Immediately follow the prompts to add your reactions, press "OK", and put the lid down.

9. After 4 minutes of incubation remove the reaction(s) from the AmpliFire. Quickly mix, spin, and reinsert the reaction(s) into the AmpliFire to continue monitoring results. Take care to ensure the tubes are in their original positions and orientations.

10. The test lasts 20 minutes and the results will be visible on the screen, and should be interpreted as follows: Blue curve = FAM = PMTV. Red curve = CalRed = Internal control. (+) = Positive for PMTV (-) = PMTV not detected (!) = Invalid

11. Note: Adapted and modified from the manual of Agdia AmplifyRP® XRT for PMTV Rapid RNA Amplification Test Kit, Product No. XCS 12501
